# Coralline algal metabolites induce settlement and mediate the inductive effect of epiphytic microbes on coral larvae

**DOI:** 10.1038/s41598-018-35206-9

**Published:** 2018-12-03

**Authors:** Luis A. Gómez-Lemos, Christopher Doropoulos, Elisa Bayraktarov, Guillermo Diaz-Pulido

**Affiliations:** 1 School of Environment and Science, and Australian Rivers Institute– Coast & Estuaries, Nathan Campus, Griffith University, 170 Kessels Road, Nathan, QLD 4111 Australia; 2 CSIRO Oceans and Atmosphere, Dutton Park, QLD 4102 Australia; 3 Centre for Biodiversity and Conservation Science, The University of Queensland, St Lucia, QLD 4072 Australia; 4 Australian Research Council Centre of Excellence for Coral Reef Studies, Townsville, Australia

## Abstract

Settlement of invertebrates is a key process affecting the structure of marine communities and underpins the ability of benthic ecosystems to recover from disturbance. While it is known that specific crustose coralline algae (CCA) are important for settlement of some coral species, the role of algal chemical compounds versus surface microbial biofilms has long been ambiguous. Using a model system - a CCA of a genus that has been shown to induce high levels of settlement of *Acropora* corals (*Titanoderma cf*. *tessellatum*) and an abundant coral species (*Acropora millepora*)- we show that chemical effects of CCA are stronger than those from CCA surface microbial biofilms as drivers of coral settlement. Biofilms contributed to some extent to larval settlement via synergistic effects, where microbial cues were dependent on the CCA primary metabolism (production of dissolved organic carbon). We propose that optimal coral settlement is caused by complex biochemical communications among CCA, their epiphytic microbial community and coral larvae.

## Introduction

Recruitment of sessile organisms is a crucial ecological process that impacts the structure and maintenance of marine communities through the successful dispersal and settlement of mobile propagules^1^. Larval settlement is a vital step in the life cycle of many invertebrates such as reef-building corals, as settlement defines the transition of motile larvae from the water column to the seafloor^2–5^. Swimming larvae meticulously test substrates prior to settlement because microhabitat selection is imperative for the post-settlement survival of newly settled recruits^6–8^. Thus, a key bottleneck to the recovery of decimated coral populations is the ability of larvae to successfully settle and recruit to optimal substrata^9–11^. It is well documented that particular species of crustose coralline algae (CCA, a group of calcifying red algae) are the preferred settlement substrate for numerous coral species^8,12–14^. Therefore, any disruption to CCA-coral settlement interactions is of particular concern to recruitment success. There is some evidence demonstrating that ocean acidification^15^, elevated sea temperatures^16^, reduced grazing^17,18^ and poor water quality^19^ alter the ecological interactions required for optimal coral settlement onto CCA. Although important progress has been made in the field of coral settlement – CCA interactions and in the nature of the chemical^8,14^ and microbiological^20–22^ cues for settlement, little is known about how these cues interact to facilitate larval settlement.

**Figure 1:**
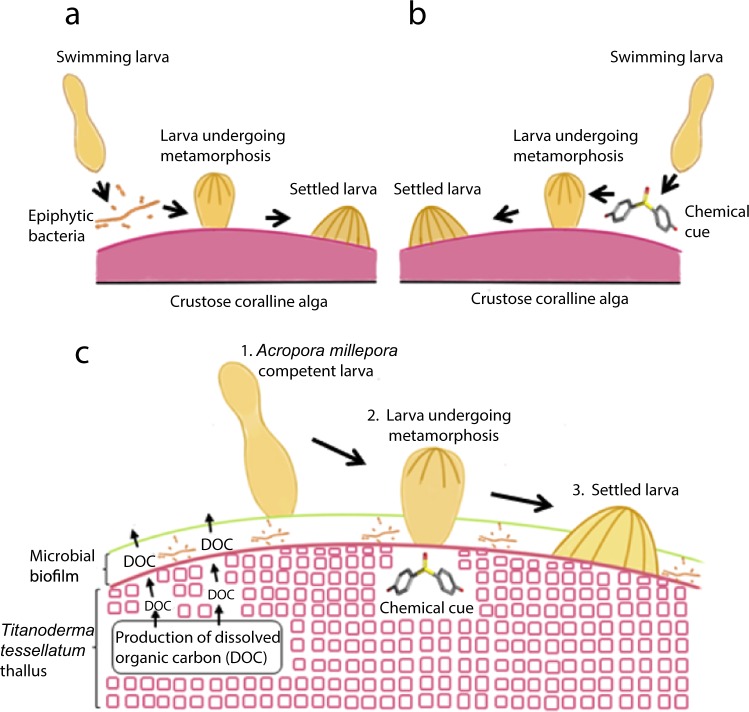
Proposed mechanisms of coral larval settlement induction facilitated by crustose coralline algae (CCA). (**a**) Larval settlement induced by CCA-epiphytic bacteria^20–22,30^. (**b**) Larval settlement induced by CCA-morphogens^7,14,34,35^. (**c**) In this study we suggest that coral settlement is the result of a synergistic interaction between CCA-chemical compounds (i.e. morphogens) and the CCA-epiphytic microbial biofilms. (1) Initially, *Acropora millepora* larva senses the epiphytic microbial biofilm. The positive effect of CCA-associated bacteria on *A*. *millepora* settlement is mediated by the release of primary metabolites (dissolved organic carbon - DOC) by the CCA *Titanoderma cf*. *tessellatum*. (2) Secondly, *A*. *millepora* larva undergoes metamorphosis after establishing direct contact with *T*. *tessellatum*; being that, the coral settlement cue produced by *T*. *tessellatum* is bound to the algal thallus due to its nonpolar nature. (3) Finally, the *A*. *millepora* larva successfully settles on the surface of *T*. *tessellatum*.

Interspecific interactions between coral larvae and CCA can be mediated by mechanisms originated in the algal thallus or by mechanisms associated with microbial communities (including bacteria) living on the surface of the algal crust. Coral larvae actively explore the benthos and are capable of sensing these cues to find suitable settlement substrate at a very fine scale^23^. Not all species of CCA facilitate larval settlement and there are in fact several CCA species that are actively avoided by coral larvae^8,12^. For those CCA species that facilitate settlement, intrinsic factors in CCA driving coral settlement are mainly of chemical nature^14,24^, although there is evidence suggesting that spectral cues (e.g. CCA colour) may also be important for settlement^25^. Other unknown factors of the CCA such as the type of skeletal mineralogy, photosynthetic pigments, and/or changes in oxygen and pH of the boundary layer due to CCA metabolism, may influence the settlement behaviour of coral larvae as well. Multiple studies have shown that corals from Caribbean and Pacific reefs, that brood or spawn their offspring, are induced to settle by contact with non-polar compounds derived from the CCA thalli^8,26,27^. However, brooding and spawning corals have also been shown to settle in response to microbial biofilms living on the surface of CCA^28–30^. Hence, despite the considerable advances in our understanding of the ecology of coral larvae and the role of CCA as inducers of coral larval settlement, it remains unclear whether coral settlement is mediated by intrinsic (i.e. compounds produced by CCA) or extrinsic (i.e. surface microbial community) or both properties of CCA (Fig. 1a,b). Further, interactions between intrinsic and extrinsic factors may exacerbate or inhibit the strength of substrate preference by coral larvae (Fig. 1c), yet complex interactions between the intrinsic and extrinsic components of CCA have not been investigated previously.

In this study, we conducted a series of manipulative laboratory experiments to elucidate the mechanisms by which a settlement stimulating CCA (*Titanoderma cf*. *tessellatum*) induces the settlement of coral larvae (*Acropora millepora*) in the Great Barrier Reef, Australia. We specifically focused on the effect of, and interactions between, intrinsic factors such as (primary and secondary) metabolites synthesized by CCA, and extrinsic factors such as CCA epiphytic microbial biofilms on coral larval settlement. Primary metabolites include products from the basic algal metabolism (e.g. photosynthesis) that are directly released to the environment and are waterborne, for instance dissolved organic carbon (DOC, mainly carbohydrates); there are however some primary metabolites produced by the algae which are not released to the environment. Algal DOC may be an important energy source for microbial biofilms and/or coral larvae (e.g. as suggested for mussel larvae by Barnard *et al*.^31^). Secondary metabolites, on the other hand, include hydrophobic compounds produced by the algae which can remain on the CCA surface and can have detrimental or beneficial effects on certain organisms^31,32^. Our findings suggest a positive role of CCA-chemical compounds as drivers of coral larval settlement in a common reef coral, while the CCA epiphytic microbial communities play a complementary role in the induction of larval settlement, which is reliant on the DOC supplied by CCA. Understanding the ecological mechanisms driving early life history stages is fundamental to comprehend the processes that facilitate the replenishment of coral populations, which is particularly important in the context of the current degradation of coral reefs world-wide^33^.

## Results

**Figure 2:**
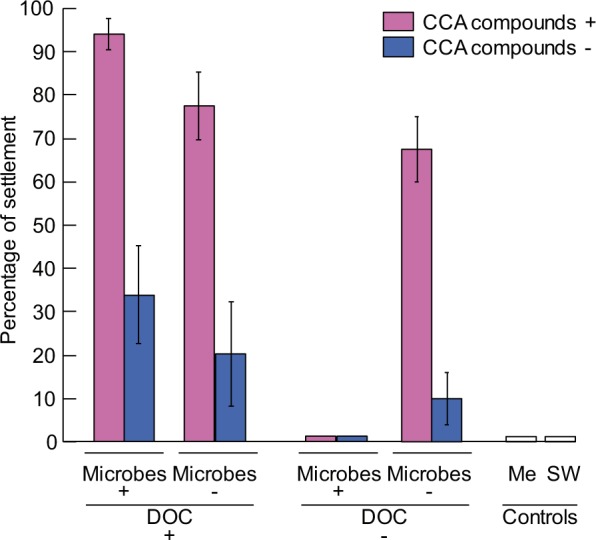
Effects of compounds produced by the crustose coralline alga (CCA) *Titanoderma cf*. *tessellatum* and associated microbes on settlement (%) of *Acropora millepora* larvae. The experimental controls were: methanol control (Me) and filtered seawater (SW). For details of the experimental treatments see Fig. 3. Abbreviations: dissolved organic carbon (DOC). Data are means ± SE (n = 5).

Experimental results revealed complex interactions among chemical extracts, DOC, and microbes (Table 1: significant interactions among three factors: three-way ANOVA n = 5, *F* = 8.01, *p* = 0.008. Table S1: two-way ANOVA n = 5, *F* = 33.43, *p* = <0.001. Table S2: two-way ANOVA n = 5, *F* = 5.50, *p* = 0.032). Despite the complexity in the interactions, chemical effects of *T*. *tessellatum* on coral larval settlement were positive and stronger compared to the surface microbial effects. Absence of chemical extracts from experimental CCA fragments significantly reduced larval settlement from 94% to 34% (64% decline, ANOVA n = 5, F = 32.36, p = 0.016) and from 77 to 20% (74% decline, ANOVA n = 5, F = 32.36, p = 0.074) in treatments with microbes present and with microbes reduced (antibiotics added), respectively (Fig. 2). In comparison, the manipulation of surface microbial abundance only had a minor, non-significant effect on settlement compared with the *T*. *tessellatum* chemical treatments. When microbial abundance was reduced with antibiotics, settlement declined only by 18% (from 94 to 77%, ANOVA n = 5, F = 32.36, p = 0.886) and 41% (from 34 to 20%, ANOVA n = 5, F = 32.36, p = 1.000) in treatments with and without chemical extracts, respectively (Fig. 2). Chemical extracts from *T*. *tessellatum* alone also induced high levels of settlement (67%), compared to the same treatment where extracts were not added (10% settlement, an 85% reduction of settlement; Fig. 2, ANOVA n = 5, F = 32.36, p = 0.005). Under one combination of treatments, however, the addition of extracts to the dead *T*. *tessellatum* fragments did not have a significant effect on coral settlement, and this occurred when microbes were added in the absence of DOC (Fig. 2, ANOVA n = 5, F = 32.36, p = 0.980). Larval settlement in the methanol control (that contained no chemical extracts) was not significantly different from that of the dead CCA (Table 2, Dunnett test, p = 0.157), indicating that the solvent had no significant effect on larval settlement behavior.

**Table 1:** Three-way ANOVA to test for the effects of chemical compounds, dissolved organic carbon (DOC) and surface microbial biofilms of the crustose coralline alga *Titanoderma cf*. *tessellatum* on settlement of larvae of *Acropora millepora* corals.

Source of variation	Df	MS	*F*	*p*
**% Larval settlement**				
Microbes	1	0.31	7.28	0.011
Chemicals	1	3.09	71.09	<0.001
DOC	1	3.04	69.82	<0.001
Microbes*Chemicals	1	0.27	6.34	0.017
Microbes*DOC	1	1.97	45.31	<0.001
Chemicals*DOC	1	0.41	9.50	0.004
Microbes*chemicals*DOC	1	0.34	8.01	0.008
Error	32	0.04		

As significant interactions occurred among treatments, further two-way and one-way ANOVAs were conducted within treatment combinations (see Supplementary Information, Tables S1, S2 and S3).

**Table 2:** One-way ANOVA to test for the effect of experimental treatments on the settlement of *Acropora millepora* larvae.

Source of variation	Df	MS	*F*	*p*	Conclusion (Dunnett test)
**Comparison of treatments against negative control (SW)**					
Treatment levels	8	1.35	38.98	<0.001	1 > 9, 2 = 9, 3 > 9, 4 = 9, 5 > 9, 6 > 9, 7 = 9, 8 = 9
Error	40	0.03			
**Comparison of treatments against methanol control (Me)**					
Treatment levels	8	1.35	38.98	<0.001	1 > 10, 2 = 10, 3 > 10, 4 = 10, 5 > 10, 6 > 10, 7 = 10, 8 = 10
Error	40	0.03			

Experimental treatments were compared using the Dunnett *post-hoc* test to determine significant differences between treatments with respect to a negative control (filtered seawater) and against the methanol control. Treatments: (1) CCA compounds, microbes and DOC, (2) CCA compounds and microbes, (3) microbes and DOC, (4) microbes, (5) CCA compounds and DOC, (6) CCA compounds, (7) DOC, (8) dead CCA (without CCA compounds, microbes and DOC), (9) negative control [filtered seawater (SW)], (10) methanol control (Me). Abbreviations: dissolved organic carbon (DOC), crustose coralline algae (CCA).

Although microbial effects on settlement were weaker compared to effects of *T*. *tessellatum* chemical extracts, microbial effects strongly depended on the availability of DOC. When DOC was naturally present or artificially supplied to treatments with dead CCA fragments, microbial biofilms induced variable levels of larval settlement (94% and 34% under presence or absence of chemical extracts, respectively, as detailed above, Fig. 2). However, when DOC was not supplied to wells containing dead CCA fragments, addition of microbial biofilms to the (dead) *T*. *tessellatum* fragments did not prompt coral settlement, independently of the presence or absence of *T*. *tessellatum* extracts (Fig. 2). This indicates that the effects of microbes are mediated by algal DOC.

In general, the presence of naturally occurring DOC and the supply of a proxy for coralline algal DOC enhanced coral larval settlement. This positive effect, as with the previous drivers, was strongly dependent on the levels of the other factors examined. In particular, DOC in the presence of surface microbes induced moderate to high levels of settlement, especially in the presence of *T*. *tessellatum* chemical extracts. However, absence of algal DOC completely cancelled the positive effect of microbes on settlement; larvae in this treatment (with microbes added and no DOC) were not induced to settle (Fig. 2; Table S1: two-way ANOVA n = 5, *F* = 33.43, *p* = <0.001), but were still actively swimming (Fig. S1 in Supporting Information).

## Discussion

The mechanisms that mediate interspecific interactions are important issues in ecology generally, and this is particularly relevant when addressing processes such as settlement and recruitment, which are fundamental to population and community structure. More specifically, the mechanisms by which some crustose coralline algae (CCA) induce the settlement of reef building corals have been debated^14,26,34^. One hypothesis proposes that coral larval settlement is induced by specific compounds (e.g. morphogens) produced by the algae^14,22,35,36^, while other studies suggest that coral settlement is driven by cues from surface-associated microbial biofilms^28–30^. These contentions need not be mutually exclusive, but our experimental results suggest that the chemical stimulatory effects of the coralline alga *Titanoderma cf*. *tessellatum* (genus shown to induce high levels of coral settlement in the Pacific and Caribbean^8,37^) are stronger than the effects of surface microbes as drivers of larval settlement of *Acropora millepora* corals. We also show, however, that surface microbial biofilms contribute to some extent to larval settlement and, importantly, induction is further stimulated by DOC exudates typical of red algae (i.e. DOC in the form of waterborne sugars). We suggest that settlement of a dominant coral species is predominantly driven by CCA-chemical compounds, but is also mediated by complex interactions and synergies between intrinsic and extrinsic (CCA-epiphytic bacteria) mechanisms of the coralline algae.

The role of CCA- chemical compounds as inducers of coral settlement has been proposed for more than 30 years when it was discovered that CCA were a key group of benthic organisms that induce settlement of marine invertebrate larvae, including corals and abalones^34,35,38^. However, the role of CCA- chemical compounds had not been independently demonstrated from the cues produced by surface microbial biofilms^7,8,34,35,38^. The study by Tebben *et al*.^14^ was the first to clearly indicate that coral settlement was triggered by CCA- morphogens, showing that algal chemical cues induced identical rates of larval settlement as live CCA. The authors also showed that the removal of bacterial communities did not alter coral settlement rates. Our results confirm a positive effect of cues originating from chemical mechanisms of the CCA on coral larval settlement. We also provide novel information by resolving the complexity of interactions between the cues from CCA- chemical compounds and surface microbial biofilms, suggesting that these two mechanisms act multiplicatively on coral settlement. Since the bioactive compound extracted in our experiment is hydrophobic (Gómez-Lemos *et al*. forthcoming), larvae must establish contact with the CCA surface (epithallus) to sense the cues and be induced to settle (Fig. 1c). This effect of CCA-compounds and biofilms, however is strongly dependent on CCA dissolved organic compounds (waterborne primary metabolites), suggesting an important synergy between the primary and secondary algal metabolism and epiphytic microbial biofilms as drivers of coral settlement induction. It is also possible that some of the effects of CCA on coral larval behaviour are related to other factors of the live CCA including colour (e.g.^25^), or changes in pH in the boundary layer due to algal metabolism. Since we are unable to tease apart the effect of colour or metabolic activity of live CCA from that of chemical compounds, biofilms and DOC, the relative contribution of these factors to larval settlement warrants further study.

Synergistic interactions between coral settlement cues produced by microbial biofilms and coralline algal DOC had not been documented in the literature, although the important role of bacteria (e.g. *Pseudoalteromonas*^21^) and their compounds^22^ on coral settlement is well known^29,30,39,40^. In our study, *T*. *tessellatum* surface microbial biofilms did not induce larval settlement when tested in isolation; however when the experimental microbial biofilms on dead *T*. *tessellatum* were supplied with a proxy for red algal DOC, microbial biofilms induced coral settlement, although not at the levels induced by chemical extracts from the alga. Thus, the effect of microbial biofilms on coral larval settlement interacts synergistically with the production of CCA waterborne metabolites (i.e. DOC). Primary producers such as CCA exudate variable amounts of photosynthetic products (photosynthates) in the form of DOC^41,42^, which are transported through water and are known to stimulate the activity and growth of microbial communities in reefs^42,43^. It has been demonstrated that four of the five carbohydrates used in our settlement assays as a proxy of red algal DOC (galactose, glucose, mannose and xylose, ranked from the highest to the lowest concentration within natural and artificial DOC), facilitate increased rates of bacterial growth on reef biofilms^44^. Johnson and Sutton^44^ found that bacteria require soluble compounds from the CCA *Lithothamnium pseudosorum* (likely *Lithothamnion proliferum*) to produce the settlement inducer for larvae of the crown-of-thorn starfish and suggested that bacteria are only inductive if they have access to algal-derivate substrates. The specific pathways by which DOC mediates the action of bacteria as inducer of coral larval settlement cannot be deduced from our experiment. However, it is likely that DOC exudates released by the CCA primary metabolism promote microbial communities that are beneficial for coral larval settlement, potentially providing the precursors required for the synthesis of bacterial morphogenic signalling substances. Barott *et al*.^45^ suggested complex interplays between DOC, CCA and adult corals as important mediators of space competition between corals and benthic algae. The reasons behind the lack of larval settlement induction by the CCA microbial biofilms in the absence of DOC (Fig. 2) are unclear, but may include differences in microbial communities between experimental treatments. Further, since CCA not only release dissolved organic matter but also harbour a bulk fraction of poorly soluble organic matter on their surfaces, the type of dissolved organic matter added (or the lack of some compounds) in the experiment may have altered the nature of the surface bacterial biofilms. Future microbiome analyses as well as detailed characterization of the dissolved and poorly soluble organic matter produced by CCA could shed light into the processes influencing the interactions between microbes, DOC and CCA. We conclude that the role of *T*. *tessellatum* microbial biofilms in the settlement of *A*. *millepora* corals is minor compared to the role of CCA- chemical compounds, and the settlement inductive effect of these microbes is mediated by primary metabolites exuded by the algae.

An unexpected result in our study was the absence of *A*. *millepora* settlement when surface microbial biofilms (obtained from living *T*. *tessellatum*) were incubated on dead *T*. *tessellatum* crusts previously impregnated with *T*. *tessellatum* extracts (Fig. 2). This result suggests that the exposure of microbial biofilms to *T*. *tessellatum* extracts may have promoted an atypical and adverse microbial community for larval settlement. It is likely that the CCA chemical extracts (which where dissolved in the methanol solvent) added to dead CCA fragments, contained both surficial (e.g. epithallial/perithallial tissue) as well as more internal chemical compounds (e.g. hypothallial tissue). Some of the extracted compounds may not be present on the surfaces of *T*. *tessellatum* in natural conditions. Consequently, microbes may have interacted with *T*. *tessellatum* internal compounds extracted with methanol, negating any positive inductive effects of CCA chemicals on larval settlement. Although larvae in this experimental treatment were actively swimming in the water column (see Fig. S1 in supplementary material), suggesting that this adverse effect only occurred at the CCA surface level. Future studies should confirm the nature of the microbial communities and the influences of internal CCA compounds on microbial communities and their effect on coral larval settlement.

Our study has provided experimental evidence unravelling the biochemical and microbial mechanisms that mediate the induction of coral larval settlement by a tropical CCA. An important settlement induction pathway is mediated by chemical cues associated with the algal tissue. Microbial biofilms contributed to a lesser extent to coral settlement via a multiplicative effect. These interactions are, however, mediated via synergistic effects triggered by the release of dissolved organic compounds from the CCA thalli. Further studies are needed to examine the role of DOC production by CCA, as well as the contribution of poorly soluble organic matter on the CCA surfaces in regulating the CCA associated microbial community. Also, the effect of other factors of the CCA, such as colour and/or changes in pH due to CCA metabolism, should be discerned from those of chemical compounds on larval attachment and metamorphosis. Successful coral settlement seems to depend on the effective functioning of the coralline alga holobiont (CCA and associated microbial community). Ecological processes that influence CCA physiology and abundance (e.g. light availability, ocean acidification^15^, water quality^46^, coral-seaweed competition^45,47^, herbivory^48^) may alter the direction and strength of coral settlement induction by *T*. *tessellatum*. Knowledge underpinning the mechanisms driving coral larvae-microbes-plant interactions and the influences of the environment on early life-history processes of coral larvae, will facilitate a better understanding of the drivers of reef recovery following disturbance.

## Materials and Methods

### Experimental approach and sample collection

**Figure 3:**
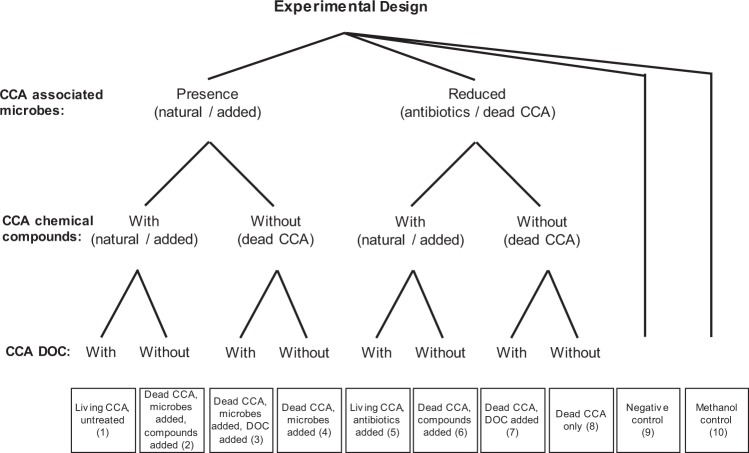
Multifactorial experimental design used in this study to test for the effects of crustose coralline algae (CCA) surficial microbial biofilms, CCA chemical compounds, and CCA dissolved organic carbon (DOC) on coral settlement. We used the CCA *Titanoderma cf*. *tessellatum* and the branching coral *Acropora millepora* for the experiment. The microbial biofilm treatment had two levels: A) presence of microbes (achieved by either using a natural, unmanipulated microbial communities present on the surface of *T*. *tessellatum*, or by experimentally adding microbial communities obtained from the surface of living *T*. *tessellatum* to dead *T*. *tessellatum* fragments), and B) reduced microbial biofilms (achieved by either adding antibiotics or using dead *T*. *tessellatum* fragments). The chemical compounds treatment (mainly hydrophobic compounds) had two levels: A) with compounds [achieved by either using unmanipulated *T*. *tessellatum* which naturally produce chemical compounds, or by adding chemical compounds extracted from the surficial thallus of *T*. *tessellatum* using methanol, and B) without compounds (using dead *T*. *tessellatum* fragments). The CCA DOC treatment had two levels: A) with DOC (achieved by either using *T*. *tessellatum* fragments that naturally release DOC, or adding a proxy for the DOC produced by *T*. *tessellatum*), and B) without DOC, obtained by using dead CCA fragments. The design included a negative control (only seawater, no CCA factors) and a procedural control (methanol control, which included a dead CCA but no CCA compounds). Numbers in brackets indicate the treatment combination used in Table 2.

We tested the independent and combined effects of intrinsic and extrinsic mechanisms of crustose coralline algae (CCA) on coral larval settlement. To do this we examined the contribution of three key factors in isolation and in combination (i.e. an orthogonal experimental design) on settlement. The three factors were: (1) chemical compounds from CCA tissue, and (2) dissolved organic carbon (DOC) released by CCA, as intrinsic mechanisms of the algal substrate, and (3) microbial biofilms associated with the CCA surface (extrinsic mechanism) (Fig. 3). Each factor had two treatment levels (see details in the experimental treatments section below). Experiments were conducted in Heron Island Research Station (HIRS) located in the southern Great Barrier Reef (GBR) during the Austral spring/summer (November and December) of 2014.

The CCA *Titanoderma cf*. *tessellatum* (Lemoine) Woelkerling, Chamberlain & Silva was chosen as a model organism because species of the genus *Titanoderma* [e.g. *Titanoderma prototypum* (synonym of *Lithophyllum prototypum* (Foslie) Foslie] are potent settlement inducers for larvae of corals of the genus *Acropora*^8,49^. *T*. *cf*. *tessellatum* is currently regarded as a synonym of *Lithophyllum prototypum* (www.algaebase.org), however, the type specimen of *L*. *prototypum* is from the Caribbean and since there are no taxonomic and molecular studies of *Titanoderma* in the GBR, we prefer to use the name *T*. *cf*. *tessellatum*. Specimens of *T*. *cf*. *tessellatum* were collected from the reef slope (between 3 and 8 m depth) at Heron Island (23°26.24S, 151°55.23E) using hammer and chisel and immediately transported to the HIRS where they were maintained in flow-through aquaria until experimental onset. The coral species *Acropora millepora* was chosen as a model organism of the coral counterpart since it is an important reef builder and commonly found in the GBR and Indo-Pacific shallow reefs^50^.

### Larvae rearing

To obtain coral larvae required for the settlement experiment, six gravid colonies of *A*. *millepora* were located and tagged on the Heron Island reef flat (23°26.53S, 151°54.98E) five days prior to predicted spawning. The tagged colonies were collected on the day of full moon and transported to the outdoor aquarium facilities at HIRS where they were kept in 600-L flow-through tanks. Spawning occurred on the 10^th^ of November 2014 at 21:30; on that day, water flow was turned off after sunset and some water was emptied from the tanks to avoid losing gamete bundles during the spawning. Egg-sperm bundles were broken apart by carefully agitating the water in the tanks, and gametes from the six different colonies were collected for cross-fertilization. Fertilization was confirmed under a dissecting microscope after 2 h. Embryos were collected and reared in 300-L sumps with ambient seawater lightly aerated under indoor laboratory conditions at 25 °C. Seawater was filtered (0.2 µm pore size) and sterilized using an ultra violet light (Aqua pro UVT 108 – 8 W) to avoid bacterial outbreaks during larval rearing. Half of the seawater volume in the rearing sumps was changed every 6 h for the first 48 h, and every 12 h thereafter to remove unfertilized eggs and dead larvae that may contaminate the rearing sumps. The larvae developed cilia and began swimming 4 days after spawning. Larvae were fully competent 11 days following spawning, upon which they were used in the settlement assays.

**Figure 4:**
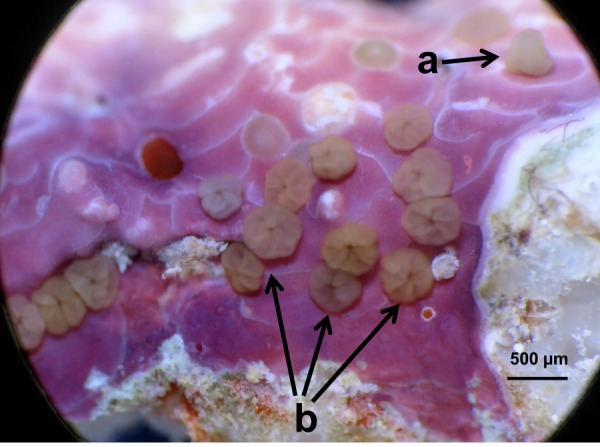
Settlement of larvae of the experimental coral *Acropora millepora* on the crustose coralline alga (CCA) *Titanoderma cf*. *tessellatum*. (**a**) 10 day old larva attached to the CCA thallus. (**b**) Larvae successfully settled, i.e. attached and metamorphosed.

Thirty *Acropora millepora* larvae were randomly selected and allocated to each experimental treatment (see below). We used plastic multi-well plates of six wells per plate. We arranged one treatment per plate to avoid cross-contamination among treatments, and the multi-well plates were located randomly on the laboratory bench. Each well contained 30 mL of seawater in a ratio of 1 larva per mL, and larvae were allowed to settle for two days (i.e. attach and metamorphose^40^, Fig. 4). Each experimental treatment had five replicate containers. All fragments of *T*. *tessellatum* used in the settlement assays had a standardized surface area of 1 cm^2^. Half the seawater in the experimental containers was carefully changed after 24 hours and any dead larvae removed.

## Experimental Treatments

### Effects of CCA compounds on larval settlement (factor 1)

To test the effect of CCA- chemical compounds on larval settlement we exposed coral larvae to two levels of this treatment, one containing tissue chemicals from the CCA *T*. *tessellatum* and the other without CCA tissue chemicals. The treatment level with algal chemical compounds was achieved by two means, adding chemical extracts obtained from the CCA *T*. *tessellatum* to dead CCA fragments (thus minimising the effects of biofilms and DOC), and using living CCA *T*. *tessellatum* (which included the effects of biofilms and DOC; Fig. 3). A further treatment using dead CCA with addition of chemical extracts + microbial biofilms + DOC could have been useful to discern the role of CCA compounds from CCA colour (e.g.^25^), but we were unable to include this treatment in our study. The treatment level without CCA compounds consisted of dead CCA fragments with no addition of CCA chemicals. Before extraction of CCA compounds, living fragments of *T*. *tessellatum* were treated with antibiotics (see details below in factor 3) to minimize the influence of compounds from CCA-associated bacteria during the chemical extraction. Following Harrington *et al*.^8^, the living surface tissue of five 1 × 1 cm fragments of *T*. *tessellatum* was scraped using scalpels and immersed in methanol (reagent grade), allowing the slurry to sit in darkness at ambient temperature for 24 h. The slurry was passed through 0.45 μm nylon filters (Sigma Aldrich®) and the filtrate retained. The filtrate was evaporated to dryness using a rotary evaporator at 40 °C. The dried extract was dissolved again in methanol (reagent grade) and 5 mL of this solution was added to each 1 × 1 cm fragments of dead *T*. *tessellatum* at the natural concentration of the CCA-chemical compounds. These fragments of *T*. *tessellatum* were allowed to dry until the methanol was completely evaporated before placing them in the experimental wells, which contained seawater; 30 larvae were then added to each of the 5 wells. Dead fragments of *T*. *tessellatum* were obtained by drying live CCA in an oven at 60 °C for 72 h and treated with antibiotics before addition of CCA extracts. A methanol control was included to test for possible effects of the solvent and extraction protocol on larval settlement (i.e. procedural control). The same volume of methanol (5 mL) used to test the CCA-chemical compounds was added to dead *T*. *tessellatum* fragments (1 × 1 cm) and allowed to dry until the methanol was evaporated. We were unable to include this procedural control into a fully factorial design because of logistical reasons, however, statistical analyses showed the methanol did not affect settlement.

### Effects of algal DOC on larval settlement (factor 2)

The DOC treatment aimed to isolate the role of DOC produced by CCA (waterborne compounds) from less polar CCA-chemical compounds, as well as from the microbial biofilms associated with the CCA surface on coral larval settlement. This treatment had two levels, with DOC or without DOC (Fig. 3). The treatment containing DOC was achieved in two ways: using untreated CCA (with natural DOC properties and quantities), or by adding artificial DOC (see below). The level without DOC was attained by using dead CCA fragments (killed as described above). Given the lack of information on the nature of the DOC from *T*. *tessellatum*, we used generic DOC constituents derived from tropical red algae as a proxy for DOC produced by CCA^51^. A mixture of 5 carbohydrates was used as a substitute for the daily production of DOC by red algae^51^. Laboratory grade arabinose (0.07206 mg^L−1^), galactose (0.69179 mg^L−1^), glucose (0.30266 mg^L−1^), mannose (0.33509 mg^L−1^) and xylose (0.27924 mg^L−1^) were dissolved in sterile-filtered seawater and poured into the experimental wells containing dead *T*. *tessellatum* crusts (as described before). This procedure was repeated daily during the span of the experiment (2 days). Seawater DOC concentrations from the experimental containers were verified following standard protocols for DOC analyses (see Supplementary Methods). Results indicated that the DOC concentration used in the DOC addition treatment level was 120 μmol L^−1^, while the ambient DOC concentration was 61 μmol L^−1^. Both ambient and elevated DOC concentrations fall within the range of values typically measured on corals reefs worldwide, varying between 57 and 160 μmol L^−1 ^^52–54^. Since the methanol used to extract the CCA chemical compounds is a polar solvent (previous section), it may have also extracted compounds including some types of sugars added here externally as DOC. However, since the main constituents of algal DOC (i.e. arabinose, galactose, glucose, mannose and xylose) are easily soluble in seawater, the contribution of those sugars to coral settlement in the treatments with chemical extracts should have been negligible.

### Effects of CCA microbial biofilms (factor 3)

To investigate the role of CCA epiphytic microbes on larval settlement we compared the rates of coral settlement on CCA across treatments that had two levels of CCA associated microbes and controls (positive and negative). The first treatment level aimed at reducing the abundance of CCA associated microbes. To do this, fragments of living *T*. *tessellatum* were treated with a mixture of antibiotics: chloramphenicol (50 mg^L−1^), tetracycline (30 mg^L−1^) and streptomycin (30 mg^L−1^) for 24 h, following Johnson and Sutton^44^. The fragments of *T*. *tessellatum* were then repeatedly (4 times) rinsed vigorously with filtered seawater and soaked in filtered seawater a fifth time for 1 h. An additional test was conducted to measure the efficacy of this antibiotic treatment (see description below). The second treatment level tested the effect of the presence of CCA-associated microbes on coral larvae settlement without the interference of CCA chemical effects. Therefore, dead fragments of *T*. *tessellatum* were incubated with microbial films obtained from living *T*. *tessellatum*. To do this, biofilms were removed from the surface of living *T*. *tessellatum* with water pressure from a waterpik® (water flosser model WP-100) using filtered-sterilised seawater and immediately added to the CCA surface. Microbes added to the surface of dead *T*. *tessellatum* were incubated at room temperature for 4 days. During incubation, the dead *T*. *tessellatum* fragments (with microbes on their surface) were kept in direct adjacent contact with thalli of living *T*. *tessellatum* to promote development of microbial communities similar to those encountered in live fragments. We did not assess the similarity of the microbial communities across treatments, thus the methodology used here to manipulate the microbial biofilms on the CCA has some limitations, which should be addressed in future studies. The positive control for the experiment consisted in untreated fragments of living *T*. *tessellatum*. A negative control was also included to test the rate of larval settlement without any settlement induction cues (neither from the CCA nor surface microbes) and this was achieved using filtered and UV-sterilised seawater only.

**Figure 5:**
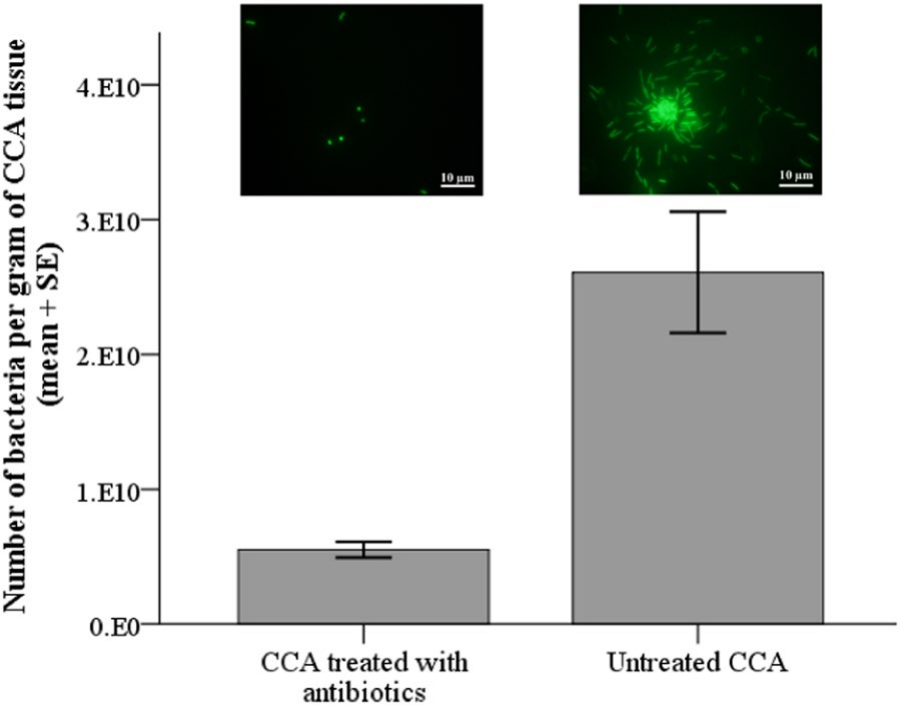
Effect of addition of antibiotics to minimize the quantity of bacteria naturally present on microbial biofilms on the surface of the crustose coralline alga *Titanoderma cf*. *tessellatum*. Insets are photographs of bacteria under epifluorescence microscopy showing a significant reduction in the number of bacteria on *T*. *tessellatum* treated with antibiotics.

The effectiveness of the antibiotic treatment used in the settlement assays, was assessed in an experiment comparing the number of bacteria living on the surface of the CCA thalli from fragments treated with antibiotics to that of fragments without the addition of antibiotics (untreated CCA; see Supplementary Methods for details of the protocol). Results of this experiment demonstrated that the antibiotic treatment used in the larval settlement assays significantly reduced the number of bacteria living on the thalli of *T*. *tessellatum* by one order of magnitude, from 2.61 + E10 (SE ± 5.32 + E09) to 5.52 + E09 (SE ± 6.91 + E08) (Fig. 5; Mann-Whitney test, *P* = 0.001, n = 7). This outcome confirmed results from previous studies that used antibiotics to reduce the abundance of bacteria associated to coral reef organisms^55,56^.

### Data analyses

A fixed factorial model for asymmetrical controls was used to test for differences in the proportion of settled larvae among treatment levels. This model was created to be applied to experiments where factors are orthogonal to one another, but with only a single group of replicated controls^57^. In our experiment, we only had one seawater control treatment (with five replicate containers) for logistical reasons, otherwise there would be a very large number of treatments and replicates to be handled at the same time while adding little information to the experiment. Likewise, there was only one group of replicated controls for the methanol addition. To use this model, first data from the treatment levels and the controls were analyzed using a one-way ANOVA followed by a Dunnett *post-hoc* test for multiple comparisons. This first step was conducted to find specific differences between the negative control of filtered seawater and the experimental treatments. This procedure (i.e. one-way ANOVA followed by Dunnett test) was also repeated to compare the experimental treatments against the methanol control (Table 2). Once those differences were stablished, larval settlement means were compared with a three-way ANOVA with CCA- chemical compounds (two levels: presence and absence of CCA- chemical compounds), CCA-associated bacteria (two levels: presence and reduction (absence) of CCA- associated bacteria), and CCA-DOC (two levels addition and no addition of synthetic CCA- DOC) as fixed factors and containers (wells) as replicates. Significant interactions occurred between treatments, therefore we conducted subsequent two-way and one-way ANOVAs within treatment combinations, followed by Tukey tests (see Supplementary Information, Tables S1, S2 and S3). Data were checked for normality using a Shapiro-Wilk test and for homogeneity of variance using a Levene’s test. Assumptions of normality and homogeneity of variance were not attained; therefore, data were Arc-sin transformed. Data from the antibiotic efficacy test were not normally distributed following transformation; consequently, the non-parametric Mann-Whitney test was used. Statistical analyses were performed using SPSS v.22.

## Electronic supplementary material

Supplementary Information

## Data Availability

The datasets generated during and/or analysed during the current study are available from the corresponding author on reasonable request.
