# Supplementary material for: Coralline algal metabolites induce settlement and mediate the inductive effect of epiphytic microbes on coral larvae
Source: Sci Rep. 2018 Dec 3;8:17557. doi: 10.1038/s41598-018-35206-9 (PMC6277392; doi:10.1038/s41598-018-35206-9)
Supplement: Supplementary file 1 — Supplementary Information [file 41598_2018_35206_MOESM1_ESM.pdf]

**Coralline algal metabolites induce settlement and mediate the inductive effect of epiphytic microbes on coral larvae**

Luis A. Gómez-Lemos, Christopher Doropoulos, Elisa Bayraktarov, Guillermo Diaz-Pulido

## Supplementary Methods

### Standard protocols for dissolved organic carbon (DOC) analyses

To verify seawater DOC concentrations from the experimental containers, seawater samples from the different DOC treatments were collected using acid-washed syringes and filtered through Polyethersulfone (PES) syringe filters. The first part of the filtered seawater was used to rinse the filter and the vial. 40 ml of filtered seawater were kept in pre-combusted glass vials (4 hours at 450°C), acid-washed Teflon cups, and preserved until analysis adding 4 drops of 85% ortophosphoric acid. Water samples for DOC analysis were stored at 4 °C and were analysed using a high-temperature catalytic oxidation technique in a TOC-VCSH Shimadzu at the Griffith University laboratories. Reference material (41 to 44 μmol DOC $L^{-1}$) provided by D. A. Hansell and W. Chen from the University of Miami was used to assess the accuracy of the estimates. Average DOC concentration of the reference material was 44.1 ± 2.9 μmol $L^{-1}$.

### Antibiotic efficacy test

To assess the effectiveness of the antibiotic treatment used in the settlement assays, we conducted an experiment comparing the number of bacteria living on the surface of the crustose coralline algae (CCA) thalli from fragments treated with antibiotics to that of fragments without the addition of antibiotics (untreated CCA). To do this we scraped off the epithallus (surficial layer) of CCA fragments (n=7 per treatment) using scalpels while

avoiding tissue desiccation to keep bacteria alive. The CCA tissue was weighed using an analytical balance (Mettler Toledo AB204-S) to standardize the number of bacteria per gram of CCA tissue. Estimating the number of bacteria per gram of tissue is more accurate than per square centimetre because the surfaces of *T. tessellatum* can be highly uneven. Epithallus from each sample of treated and untreated CCA were placed in individual 1.5 mL Eppendorf tubes with 1350 µL of filtered seawater (0.2 µm), and 150 µL of 10% methanol solution (V/V) were added to each tube to detach tissue-associated bacteria. The samples were then treated for 15 min at 35 °C in an ultrasonic bath (Ultrasonic Bath FXP14 Series 10.7L, 2 x 320 W per period) and centrifuged for 1 min at 2000 r.p.m (Sigma 1 -14) to remove detrital particles. A subsample of 50 µL of the supernatant was mixed with 6 µL of SYBR Green I (Molecular Probes-Invitrogen, Carlsbad, CA, USA) stain solution (1:100), used for rapid and accurate determination of bacterial abundances in diverse marine samples[1]. Bacteria were counted using a epifluorescence microscope at 1000X magnification (fluorescence microscope Olympus BX51, lamp: Olympus U–RFL-T) and 20 subsamples (fields of view) per replicate (n=7) examined randomly [2]. The average number of bacteria per replicate was standardized as a function of the weight of the CCA tissue (number of bacteria per gram) from which the sample was taken.

**References**


1 Lunau, M., Lemke, A., Walther, K., Martens-Habbena, W. & Simon, M. An improved method for counting bacteria from sediments and turbid environments by epifluorescence microscopy. *Environ. Microbiol.* **7**, 961-968 (2005).

2 Noble, R. T. & Fuhrman, J. A. Use of SYBR Green I for rapid epifluorescence counts of marine viruses and bacteria. *Aquat. Microb. Ecol.* **14**, 113-118 (1998).

## Supplementary Tables

**Supplementary Table S1**. Two-way ANOVA to test for the effect of microbial biofilms and CCA-chemical compounds on settlement of *Acropora millepora* larvae in treatments with and without CCA dissolved organic carbon (DOC). Two-way ANOVAs were followed by one-way ANOVAS and post-hoc Tukey tests. Abbreviations: dissolved organic carbon (DOC), crustose coralline algae (CCA).

| **Source of variation** | **Df** | **MS** | ***F*** | ***p*** |
|---|---|---|---|---|
| Treatments with DOC | | | | |
| Microbes | 1 | 0.35 | 5.17 | 0.037 |
| Chemicals | 1 | 2.88 | 42.16 | < 0.001 |
| Microbes*chemicals | 1 | 0.002 | 0.03 | 0.862 |
| Error | 16 | 0.06 | | |
| Treatments without DOC | | | | |
| Microbes | 1 | 1.93 | 103.92 | < 0.001[a] |
| Chemicals | 1 | 0.62 | 33.43 | < 0.001[b] |
| Microbes*Chemicals | 1 | 0.62 | 33.43 | < 0.001 |
| Error | 16 | 0.01 | | |

[a]: One-way ANOVA with chemicals present: p = < 0.001 (Tukey: with microbes < without microbes); One-way ANOVA without chemicals: p = 0.022 (Tukey: with microbes < without microbes).

[b]: One-way ANOVA with microbes present: p = 0.332; One-way ANOVA without microbes: p = < 0.001 (chemicals > without chemicals).

**Supplementary Table S2.** Two-way ANOVA to test for the effect of dissolved organic carbon (DOC) and CCA surface microbial biofilms in treatments with and without CCA-chemical compounds on settlement of *Acropora millepora* larvae. Two-way ANOVAs were followed by one-way ANOVAS and post-hoc Tukey tests.

| **Source of variation** | **Df** | **MS** | ***F*** | ***p*** |
|---|---|---|---|---|
| Settlement when chemicals are present | | | | |
| DOC | 1 | 2.84 | 105.96 | < 0.001[a] |
| Microbes | 1 | 0.59 | 22.04 | < 0.001[b] |
| DOC*Microbes | 1 | 1.99 | 74.06 | < 0.001 |
| Error | 16 | 0.02 | | |
| Settlement when chemicals are absent | | | | |
| DOC | 1 | 0.60 | 10.05 | 0.006[c] |
| Microbes | 1 | 0.001 | 0.01 | 0.915 |
| DOC*Microbes | 1 | 0.33 | 5.50 | 0.032 |
| Error | 16 | 0.06 | | |

[a]: One-way ANOVA with microbes present: p = < 0.001 (Tukey DOC > No DOC); One-way ANOVA without microbes: p = 0.332.

[b]: One-way ANOVA with DOC present: p = 0.050 (Tukey: with microbes > without microbes); One-way ANOVA without DOC: p = < 0.001 (Tukey: with microbes < without microbes).

[c]: One-way ANOVA with microbes: p = 0.001 (Tukey: DOC > No DOC); One-way ANOVA without microbes, p = 0.647.

**Supplementary Table S3.** Two-way ANOVA to test for the effect of CCA-chemical compounds and dissolved organic carbon (DOC) on settlement of *Acropora millepora* larvae in treatments with CCA associated microbial biofilms and with reduced CCA microbial biofilms. Two-way ANOVAs were followed by one-way ANOVAS and post-hoc Tukey tests.

| **Source of variation** | **Df** | **MS** | ***F*** | ***p*** |
|---|---|---|---|---|
| Settlement when microbes are present | | | | |
| Chemicals | 1 | 0.76 | 32.14 | < 0.001[a] |
| DOC | 1 | 4.95 | 209.20 | < 0.001[b] |
| Chemicals*DOC | 1 | 0.76 | 32.14 | < 0.001 |
| Error | 16 | 0.02 | | |
| Settlement when microbes are absent | | | | |
| Chemicals | 1 | 2.61 | 41.17 | < 0.001 |
| DOC | 1 | 0.05 | 0.90 | 0.355 |
| Chemicals*DOC | 1 | 0.001 | 0.02 | 0.885 |
| Error | 16 | 0.06 | | |

[a]: One-way ANOVA with DOC present: p = < 0.001 (Tukey: with chemicals > without chemicals); One-way ANOVA without DOC: p = 0.332.

[b]: One-way ANOVA with chemicals present: p = <0.001 (Tukey: DOC > No DOC); without chemicals, p = 0.001 (Tukey: DOC > No DOC).

**Supplementary Figure**

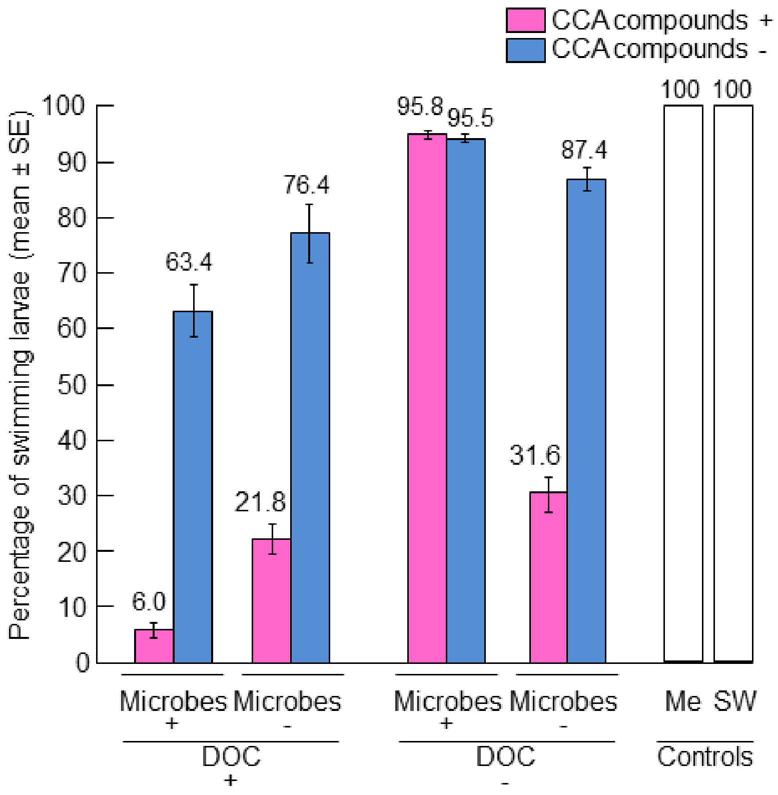


**Supplementary Figure S1.** Effects of compounds produced by the crustose coralline alga (CCA) *Titanoderma cf. tessellatum* and associated microbes on swimming larvae (%) of *Acropora millepora*. Dissolved organic carbon (DOC). The experimental controls were: methanol control (Me) and filtered seawater (SW). Data are means ± SE (n= 5). Numbers above bars indicate the actual mean value.
